# Isolation and Characterization of Nickel-Tolerant *Trichoderma* Strains from Marine and Terrestrial Environments

**DOI:** 10.3390/jof7080591

**Published:** 2021-07-23

**Authors:** Jewel C. De Padua, Thomas Edison E. dela Cruz

**Affiliations:** 1 The Graduate School, University of Santo Tomas, España Blvd., Manila 1008, Philippines; jcdp03@gmail.com; 2 Research Center for the Natural and Applied Sciences, Fungal Biodiversity, Ecogenomics and Systematics (FBeS) Group, University of Santo Tomas, España Blvd., Manila 1008, Philippines; 3 Department of Biological Sciences, College of Science, University of Santo Tomas, España Blvd., Manila 1008, Philippines

**Keywords:** agar culture, bioremediation, heavy metal tolerance, marine-derived fungi, pollution

## Abstract

Nickel contamination is a serious environmental issue that requires immediate action. In this study, 23 strains of *Trichoderma* were isolated from terrestrial and marine environments and identified using a polyphasic approach of morphological characterization and ITS gene sequence analysis. The *Trichoderma* strains were tested for their tolerance and biosorption of nickel. Our results showed the growth of all *Trichoderma* strains on Trichoderma Selective Medium (TSM) with 50–1200-ppm nickel, indicating their tolerance of this heavy metal even at a relatively high concentration. Six *Trichoderma* strains (three isolated from terrestrial substrates and three from marine substates) had the highest radial growth on TSM with 50-ppm Ni. Among these fungal isolates, *Trichoderma asperellum* (S03) isolated from soil exhibited the best growth after 2 days of incubation. For the biosorption of nickel, the accumulation or uptake efficiency by the six selected *Trichoderma* was determined in Potato Dextrose Broth (PDB) supplemented with 50-ppm Ni using a Flame Atomic Absorption Spectrophotometer (AAS). The percent uptake efficiency of the three strains of *T. asperellum* (S03, S08, and LL14) was computed to be up to 66%, while *Trichoderma virens* (SG18 and SF22) and *Trichoderma inhamatum* (MW25) achieved up to 68% uptake efficiency. Observation of the *Trichoderma* strains with Scanning Electron Microscopy (SEM) before and after the absorption of nickel showed very minimal damage on the hyphal and conidial surface morphology, but changes in the colonial characteristics were observed. Our study highlighted the potential of terrestrial and marine strains of *Trichoderma* for the bioremediation of nickel pollution.

## 1. Introduction

Heavy metals may cause damage by moving up the food chain to finally accrue in humans [1]. Moreover, due to their chemical stability, they persist in the environment. As a result, several methods have been devised for the treatment and removal of heavy metals in contaminated sites [2]. However, these traditional physicochemical processes for the remediation of polluted soils are expensive and often do not permanently alleviate the pollution hazard. In addition, health hazards are associated with heavy metal pollution [3]. Exposure by inhalation, ingestion, or skin contact can occur in heavy metal production plants, e.g., of nickel and nickel alloy, as well as in metal welding, electroplating, grinding, and cutting operations. The inhalation of nickel can cause cancer of the lungs, nose, and sinuses [4]. These all necessitate the removal of heavy metal contamination, particularly nickel, from contaminated sites. The use of living organisms such as fungi has been successfully used as adsorbing agents for the removal of heavy metals [5]. For instance, *Aspergillus niger* and *A. flavus* removed heavy metals from aqueous metal solution and metal-contaminated effluent by a bioaccumulation mechanism [6]. The reduction of six major toxic heavy metals, i.e., Cu, Zn, Pb, Cr, Cd, and Ni, by *A. niger* and *A. flavus* in effluent was found to be significantly higher as compared to industrial-treated effluent [7]. Other fungi were also used in the treatment of heavy metals and other organic-based pollutants. For example, the marine fungus *Dendryphiella salina* was used in the biosorption of mercury [8], while freshwater fungal isolates could degrade pesticides [9]. Even fungus-like protists such as slime molds or myxomycetes could be involved in the biosorption of heavy metals [10]. Interestingly, the genus *Trichoderma* are among those fungal taxa that were reported resistant to many toxic compounds, including fungicides, herbicides, and other organic pollutants and, in some cases, can degrade these toxic contaminants [11,12]. The toxic trinitrotoluene (TNT) was degraded by *T. viride* [13]. Other studies showed the ability of *Trichoderma* to degrade hydrocarbons and other organic pollutants [14,15]. Evidence has also suggested that *Trichoderma* exhibits a considerable tolerance for metals and accumulates high amounts of metals from polluted habitats, which makes *Trichoderma* become a dominant organism in some polluted environments [16]. Thus, *Trichoderma* spp. may play an important role in an eco-friendly metal removal technology and have acquired an exceptional credit as part of a sustainable approach to bioremediation [17]. It is also worth mentioning that, unlike other pollutants, heavy metals can be removed from wastewater by a biosorbent through different mechanisms, such as: (i) chemical transformations involving phase changes, (ii) bioaccumulation, which includes metabolism-dependent processes leading to the metal transport into the fungal cells, and (iii) biosorption, which is a surface mechanism that does not involve any metabolic process [11,12]. The latter mechanism is considered to be the most significant in metal removals through fungal biomass and can be attributed to ion exchange, coordination, or covalent bonding to the cell wall.

The fungus *Trichoderma* is predominantly found in many forested areas, animal manure, leaf litter, and all types of soil, including sludge [18,19]. *Trichoderma* have also been isolated from the marine environment [20] and are known for their tolerance to many toxic contaminants [21]. Based on the aforementioned facts related to the ability of *Trichoderma* species to remediate toxicants, our study was designed to isolate, characterize, and evaluate the ability of *Trichoderma* isolates from different terrestrial and marine substrates to tolerate nickel contaminants.

## 2. Materials and Methods

### 2.1. Substrates Collection

Terrestrial substrates, i.e., soil and leaf litter, and marine substrates, i.e., marine water (= seawater), sea foam, decayed seaweeds, and decayed seagrasses, were collected from different sites in Luzon Island, Northern Philippines. The soil samples were collected at Mt. Talipanan, Oriental Mindoro (13°29.241′ N; 120°53.241′ E; 223 m above sea level, masl) from a 0–15-cm depth and placed in a zip-lock plastic bag. Leaf litter collected from La Mesa Ecopark, Philippines (14°42.673′ N; 121°4.683′ E; 83 masl) and decayed seaweeds and seagrasses from the coastal areas of Sorsogon, Philippines (12°44.978′ N; 124°5.762′ E; 363 masl) were also placed in zip-lock plastic bags, while marine water and seafoam along the coast of Las Piñas—Parañaque Ecotourism Park, Philippines (14°29.998′ N; 120°59.333′ E; 12 masl) were collected on sterilized bottles. All collected samples were stored in an ice box during transport to the laboratory and were processed within 24–48 h for the isolation of *Trichoderma*.

### 2.2. Isolation of Trichoderma

#### 2.2.1. Soil

One gram of soil sample was suspended in 9-mL sterile distilled water. The soil suspension was serially diluted up to a 10^−3^ dilution level, from which 0.1 mL was spread-plated on Trichoderma Selective Medium (TSM). Trichoderma Selective Medium contained MgSO_4_·7H_2_O (0.2 g), K_2_HPO_4_ (0.9 g), NH_4_NO_3_ (1.0 g), KCl (0.15 g), rose bengal (0.15 g), glucose (3 g), and agar (20 g) dissolved in 950 mL of distilled water following the composition described by Williams et al. (2003) [22]. Three plates were used per sample. All culture plates were incubated at room temperature for 5–7 days and monitored daily for fungal growth resembling *Trichoderma*. To prevent the growth of contaminating bacteria, TSM was also supplemented with chloramphenicol (250 mg/L) and streptomycin (9 mg/L). After incubation, the colonies of *Trichoderma* were purified by point inoculation on freshly prepared TSM agar.

#### 2.2.2. Leaf Litter, Seaweeds, and Seagrasses

Initially, leaf litter and decayed seaweeds/seagrasses were rinsed with tap water to remove any adhering soil. The plant and algal samples were ground, with one gram of the powdered substrate suspended in 9 mL of distilled water. The suspension was serially diluted up to 10^−3^ and spread-plated on TSM agar and incubated as described above.

#### 2.2.3. Seawater and Sea Foam

For these samples, 0.1 mL of seawater and sea foam was directly spread-plated on freshly prepared TSM agar. The incubation and isolation of *Trichoderma* was done as previously described.

### 2.3. Polyphasic Approach to the Characterization of Isolated Trichoderma

The identification of *Trichoderma* was based on the morphological characterization and molecular method. Herein, the isolated *Trichoderma* was grown on TSM agar to prepare for Henrici slide fungal cultures. To do this, a portion of freshly prepared TSM agar was cut and placed onto a clean glass slide supported by applicator sticks inside a petri dish lined with filter paper (moist chamber). Afterwards, *Trichoderma* spores were inoculated at two sides of the TSM agar blocks and covered with a cover slip. The setup was incubated at room temperature for 2 to 3 days. Following incubation, the agar blocks were removed, and the fungal growth was covered with a cover slip and with lactophenol cotton blue as the mounting medium and then observed with a compound light microscope. The identification of *Trichoderma* was done by comparing the pigmentation present, the hyphal and conidial morphologies, patterns of branching in the conidiophores, and the presence or absence of chlamydospores. The taxonomic key provided by Bissett was utilized to identify the section from which the isolates belong [23].

Genomic DNA of selected mycelial cultures of *Trichoderma* were extracted using the benzyl chloride extraction method of Zhu et al. (1993) [24]. The extracted DNA was dissolved in Tris-EDTA (TE) buffer and amplified by PCR in a 25-μL reaction mixture composed of 2-μL DNA, 2 μL each of the forward ITS1 (5′–TCCGTAGGTGAACCTGCGG–3′) and reverse ITS4 (5′–TCCTCCGCTTATTGATATGC–3′) primer pair for six *Trichoderma* strains [25] or with the forward ITS5 (5′–GGAAGTAAAAGTCGTAACAAGG–3′) and reverse ITS4 (5′–TCCTCCGCTTATTGATATGC–3′) primer pair for the other remaining strains [26], which were difficult to amplify with the first primer pair, and 19-μL PCR mix. The PCR conditions consisted of pre-denaturation at 94 °C for 1 min and denaturation at 94 °C for 30 s, followed by 35 cycles at 55 °C for 30 s, with a final extension at 72 °C for 10 min and cooling at 4 °C. The PCR products were evaluated by gel electrophoresis and sent for the sequencing of target genes. PCR amplification and DNA sequencing was done by Macrogen (Seoul, South Korea) as an outsource service.

For species identification, the forward and reverse sequences were edited using the BioEdit 7.2.5.0 sequence alignment editor [27]. Then, it was queried in the BLAST search engine (http://blast.ncbi.nlm.nih.gov/Blast.cgi, accessed on 6 December 2016) for all published related sequences. These sequences, along with the specimens, were aligned and edited using BioEdit via the accessory application ClustalW multiple alignment. Lastly, the phylogenetic trees were constructed using MEGA Version 7 [28] based on a maximum likelihood analysis to confirm the identities of the isolated *Trichoderma*.

### 2.4. Adaptation of Trichoderma Strains to Marine Environment

To test if the isolated *Trichoderma* were adapted to the marine environment and not as simply dispersed spores or as transient fungi, the *Trichoderma* isolates were grown on culture plates with TSM and malt extract agar (MEA) without or with 35-g/L marine salt (Product Num. S9883, Sigma-Aldrich, St. Louis, Missouri, USA). Mycelial agar disks were cut approximately 5 mm from the colony margin of 3-day old *Trichoderma* cultures using a flame-sterilized cork borer and were inoculated onto the TSM/MEA culture plates. All culture plates in triplicates were incubated at room temperature, and the colony radial growth (three readings per plate) was measured from the agar disk to the margin of the colony on the 1st day up to 3rd day of incubation. The colony extension rate (CER) was computed as previously described by dela Cruz et al. (2006) [29].

$$\mathrm{CER}=\frac{\mathrm{Mean}\;\mathrm{colony}\;\mathrm{radial}\;\mathrm{growth}\;\left(\mathrm{day}\;3\right)-\mathrm{Mean}\;\mathrm{colony}\;\mathrm{radial}\;\mathrm{growth}\;\left(\mathrm{day}\;1\right)}{\mathrm{Number}\;\mathrm{of}\;\mathrm{days}\;\mathrm{of}\;\mathrm{incubation}\;\left(\mathrm{day}\;3\right)}$$

Then, a paired *t*-test was computed for the CER on TSM/MEA with and without marine salts to determine if the presence or absence of marine salt in the medium significantly affected the colony extension rates of the isolated *Trichoderma* strains.

### 2.5. Assay for Heavy Metal Tolerance of Trichoderma Strains

All *Trichoderma* isolates were tested for their nickel tolerance. TSM (pH = 4) was prepared with deionized water and supplemented with various concentrations, i.e., 50, 100, 300, 500, 700, 900, and 1200 mg/L, of nickel (supplied as NiSO_4_) salts and sterilized in the autoclave for 20 min at 121 °C (15 psi). An agar mycelial block was cut approximately 5 mm each from the colony margin of 3-day old *Trichoderma* cultures and inoculated aseptically on TSM plates supplemented with chloramphenicol, streptomycin, and with the different concentrations of nickel. The culture plates were then incubated at room temperature (22–25 °C) for up to 7 days until the *Trichoderma* isolates occupied the whole petri dish. A total of three plates were used for each treatment. Afterwards, the colony growth of the *Trichoderma* was calculated by measuring the radius in centimeter of the colony extension against the control (medium without nickel). The tolerance index (Ti) was computed, where Dt is the radial extension (cm) of the treated colony, and Du is the radial extension (cm) of the untreated colony [30]:

$$\mathrm{Ti}=\frac{\mathrm{Dt}}{\mathrm{Du}}$$

To assess the ability of the *Trichoderma* strains to grow on TSM supplemented with different concentrations of nickel in terms of their radial extension rates, the Ti was assigned as follows: (−) 0 mm, nontolerant; (+) 0.1–0.52 mm, moderately tolerant; (++) 0.53–1.04 mm, highly tolerant; and (+++) 1.05–1.56 mm, most tolerant. Isolates that showed the top/best tolerance to nickel were selected for the biosorption assay for nickel.

### 2.6. Biosorption Assay of Nickel by Trichoderma

Batch biosorption experiments were carried out for the six selected *Trichoderma* strains (three terrestrial and three marine) that showed best tolerance to nickel. In 250-mL flasks containing 100-mL Potato Dextrose Broth (PDB, pH = 4), 50-ppm nickel (as NiSO_4_) was added. Three agar plugs from the colony margin of actively growing *Trichoderma* were inoculated in each 250-mL flask (in triplicates) containing PDB and NiSO_4_ to elucidate their ability to absorb nickel ion. Each flask was kept under stationary conditions at room temperature (22–25 °C) for seven days. After incubation, the biomass was separated using a sterile Whatmann no. 1 filter paper. Subsequently, two grams of oven-dried fungal biomass from six *Trichoderma* strains was treated with a mixture of nitric and hydrochloric acids in the ratio of 2:2. The mixture was then kept on a hot plate at 80 °C until the appearance of brown color. Double-distilled water was added, and the mixture was filtered through filter paper. We used an Atomic Absorption Spectrophotometer (AAS) to analyze the filtrate for metal concentration. The percent removal efficiency of nickel by fungal biomass was calculated using the equation below, where Ci = initial concentration of metal in the solution (= 50 mg/L) and Cf = final concentration of the metal in the biomass filtrate (mg/L) [30].

$$\%\;\mathrm{removal}\;\mathrm{efficiency}=\frac{\left(\mathrm{Ci}-\mathrm{Cf}\right)\times 100}{\mathrm{Ci}}$$

For the statistical analysis of data, a *t*-test was used to determine the significant differences between treatments. Significance was reported at the 95% confidence interval (*p* value < 0.05). 

### 2.7. Evaluation of Surface Morphology of Trichoderma Grown on PDB with Nickel

The hyphal surface morphology of *Trichoderma* grown on PDB with or without 50-ppm nickel was observed with a scanning electron microscope. This is to evaluate the possible effects of nickel on the morphological traits of *Trichoderma*. The fungal biomass harvested from Section 2.6 were cut in no more than 1 cm in length and examined using the Hitachi 3000 scanning electron microscope model under a magnification of 10,000×. The digital images were generated and observed for any aberration on the hyphal surface and conidia surface ornamentation. This would give baseline information on the possible effects of nickel on *Trichoderma*.

## 3. Results

### 3.1. The Isolated Trichoderma Strains

**Figure 1:**
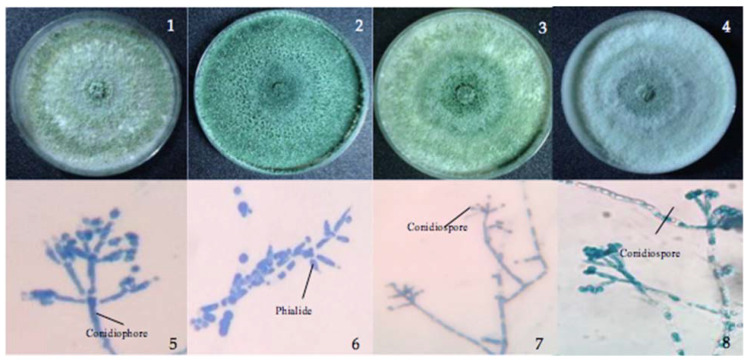
Cultures of *Trichoderma*. Colonies on TSM. (**1**–**4**) *T. asperellum, T. inhamatum, T. virens,* and *T. harzianum*. (**5**–**8**) Conidiophores, phialides, and conidia densely clustered on the wide main axis.

In this study, a total of 23 *Trichoderma* strains, i.e., nine from soil, six from leaf litter, one from seagrass, two from decayed seaweeds, four from sea foam, and one from marine water or seawater, were isolated from terrestrial and marine substrates from different sites at Luzon Island, Northern Philippines and characterized as belonging to four morphospecies (Figure 1). *Trichoderma asperellum* (12 isolates) was recorded with the highest frequency, followed by *T. harzianum* (7 isolates) (Table 1). It is known that *T. asperellum* and *T. harzianum* are widely distributed, and in this study, these two *Trichoderma* species were also obtained from varied substrates indicating their ability to thrive in both terrestrial and marine habitats. Among the species, *T. inhamatum* had the lowest number of isolates, with only one strain. Between the different substrates, soil harbored the highest number of isolates as expected, followed by leaf litter and sea foam, and with only one strain for the remaining marine substrates.

*Trichoderma asperellum* isolated from soil and leaf litter had a conidiophore with long, narrow main axes and phialides arising in whorls of three, while *T. virens* from seagrass and sea foam showed a conidial ball arising from elongated, appressed phialides from the apex of micronematous conidiophore (Figure 1). *Trichoderma inhamatum* from marine water is described with a narrow conidiophore and uncrowded, elongate phialides and a conidial ball at the apex, while *T. harzianum* from sea foam showed the same conidial ball as *T. virens* arising from appressed phialides at the apex of micronematous hyphae. Examination of the morphological characteristics provided a preliminary identification of the isolates.

**Figure 2:**
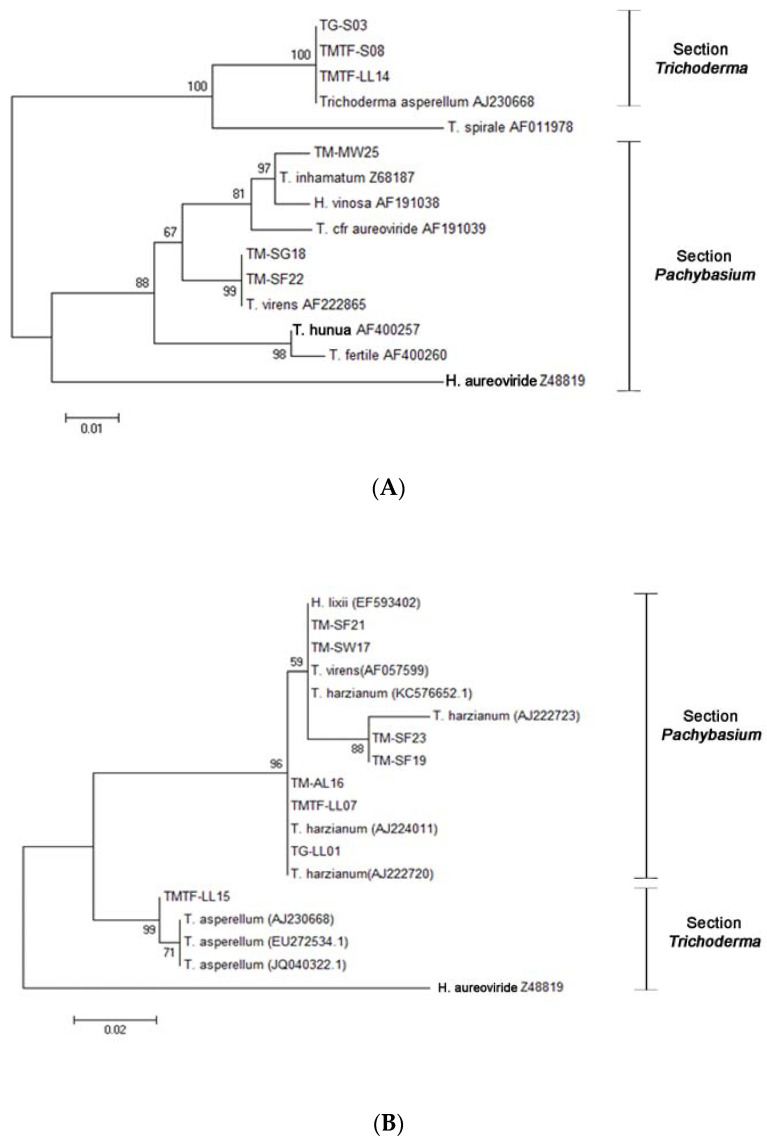
Maximum likelihood tree for the isolated *Trichoderma*: (**A**) ITS1 and ITS4 sequence analysis using the Kimura 2-parameter model. Substrates and isolate code were as follows: soil (TG-S03 and TMTF-S08), leaf litter (TMTF-LL14), marine water (TM-MW25), seagrass (TM-SG18), and sea foam (TM-SF22). (**B**) ITS5 and ITS4 sequence analysis using the Kimura 2-parameter model. Substrates and isolate code were as follows: sea foam (TM-SF21, TM-SF23, and SM-SF19); seaweed (TM-SW17); aerial leaf litter (TM-AL16); and leaf litter (TMTF-LL07, TG-LL01, and TMTF-LL15). Related sequences were obtained from GenBank and Lieckfeldt et al. [31]. *Trichoderma aureoviride* Z48819 serves as the outgroup.

An analysis of the ITS regions of the rDNA with the ITS1 and ITS4 primer pair confirmed the identities of the six *Trichoderma* strains and was well-supported by a bootstrap value above 98 within section *Trichoderma* and section *Pachybasium*. The isolated fungi were therefore identified as *T. asperellum* (TG-S03, TMTF-S08, and TMTF-LL14); *T. virens* (TM-SG18 and TM-SF22); and *T. inhamatum* (TM-MW25) (Figure 2A). However, some strains failed to produce workable sequences with the first primer pair, and hence, the ITS5 and ITS4 primer pair was used to identify these other *Trichoderma* isolates. The remaining *Trichoderma* showed a bootstrap value above 96 and were identified as *T. harzianum* (TM-SF21, TM-SF23, TM-SF19, TM-AL16, TMTF-LL07, and TG-LL01); *T. virens* (TM-SW17); and *T. asperellum* (TTF-LL15) (Figure 2B).

### 3.2. Adaptation of the Isolated Trichoderma to the Marine Environment

**Figure 3:**
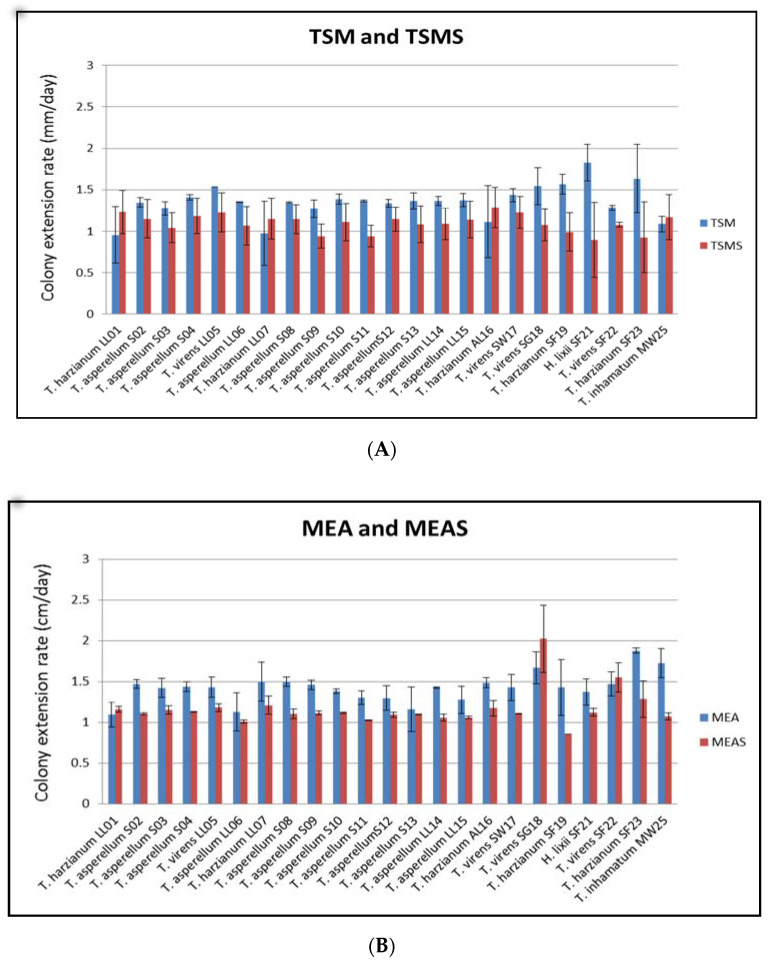
Colony extension rates of *Trichoderma* strains grown on (**A**) TSM agar and TSM agar with marine salts (TSMS) and (**B**) MEA and MEA with marine salts (MEAS). The substrate codes were as follows: leaf litter (LL), soil (S), seaweed (SW), seagrass (SG), sea foam (SF), marine water (MW), and aerial leaf litter (AL).

Most of the 23 *Trichoderma* strains, whether isolated from marine or terrestrial substrates, grew much better on TSM (19 strains) than in TSM with salt (four strains) and on MEA (20 strains) than in MEA with salt (three strains) (Figure 3). Although most of the isolates showed favorable growth in the environment without marine salt, they can still grow in the presence of marine salt with high colony extension rates. The statistical analysis by a one-tailed paired *t*-test confirmed that the mean colony extension rates in TSM and MEA were greater than TSMS and MEAS and that the difference was highly significant (*p*-value 0.0427, α = 0.05).

### 3.3. Tolerance of Nickel by Marine and Terrestrial Trichoderma

While, generally, all *Trichoderma* isolates tolerated Ni, as evident by their growth at a certain threshold, 13 terrestrial and six marine strains grew on TSM agar with 1200-ppm nickel (Table 2). Among these, *T. asperellum* (S03) isolated from soil and *T. virens* (LL05) from leaf litter exhibited the best growth after 2 days of incubation, while two marine strains, *T. harzianum* from sea foam (SF23) and *T. inhamatum* from marine water (MW25), showed a similar growth pattern. Six promising *Trichoderma* strains, identified as *T. asperellum* (S03), *T. asperellum* (S08), *T. asperellum* (LL14), *T. virens* (SG18), *T. virens* (SF22), and *T. inhamatum* (MW25), which had moderate-to-active growth at TSM with nickel even at 1200 ppm, were then chosen for the biosorption experiment.

**Figure 4:**
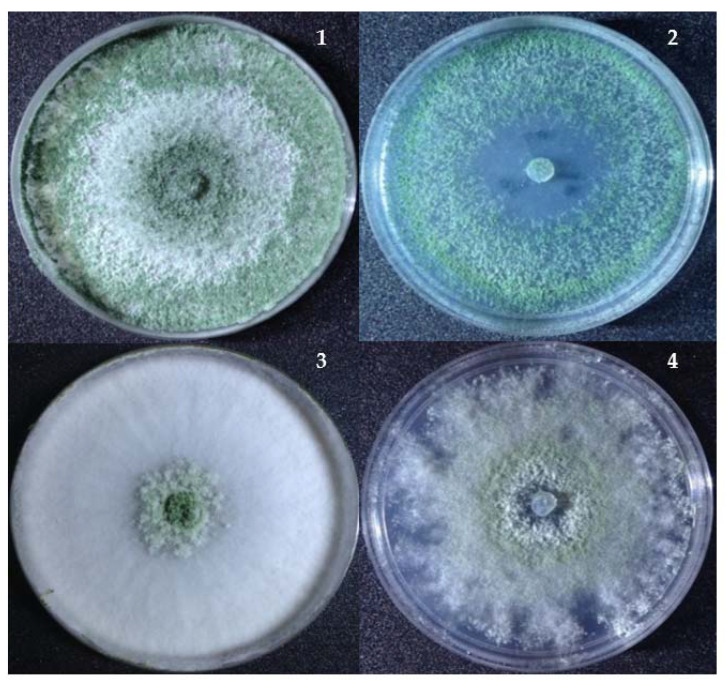
Changes in the colony morphology of *Trichoderma* at a high concentration of nickel (NiSO_4_). (**1**) and (**2**) *T. asperellum* (S12) grown on TSM without and with 500-ppm nickel and (**3**) and (**4**) *T. inhamatum* (MW25) grown on TSM without and with 900-ppm nickel.

However, the growth of *Trichoderma* at higher nickel concentrations (500–1200 ppm) resulted in changes in the colony color from dark green or pure white to light green/yellow (Figure 4). It was noticed that the colonial growth and surface morphology was significantly influenced by the nickel—in particular, at 500–1200 ppm. At 900-ppm nickel, there was a significant reduction in the radial mycelial growth for *T. inhamatum*. The effects of nickel at a higher concentration also showed a decreasing growth rate and disrupted colonial morphological appearance.

### 3.4. Effect of Nickel Exposure on the Surface Morphology of Trichoderma

**Figure 5:**
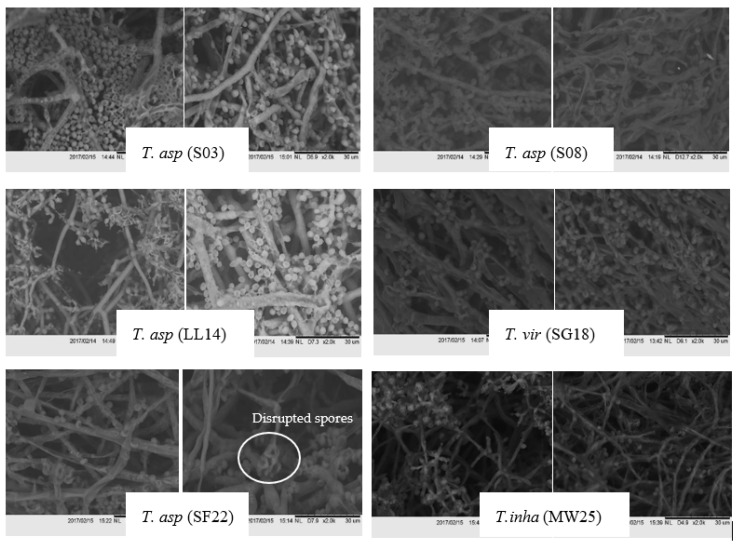
Scanning electron micrograph under a high magnification of 10,000× of the six *Trichoderma* strains before (left) and after (right) exposure to 50-ppm nickel. The isolate and substrate codes were as follows: *T. asperellum*—soil (T. asp S03/S08), *T. asperellum*—leaf litter (T. asp LL14), *T. asperellum*—sea foam (T. asp SF22), *T. virens*—seagrass (T. vir SG18), and *T. inhamatum*—marine water (T. inha MW25).

The effects of nickel exposure on the hyphal and conidial morphologies of *Trichoderma* are presented in Figure 5. The morphological alterations resulted in uneven and disrupted spores that were observed through scanning electron microscopy in treatments with 50 ppm of nickel after seven days of exposure. Moreover, aggregation of hyphae at the early stage and minimal aberrations on the conidial surface were evident. In contrast, untreated *Trichoderma* isolates showed normal fungal hyphae.

### 3.5. Biosorption Capacity of Isolated Trichoderma Strains

Table 3 shows the percent removal of nickel by the *Trichoderma* biomass. After seven days of exposure at room temperature with an initial nickel concentration of 50 ppm, *T. asperellum* (S03), *T. virens* (SG18), and *T. inhamatum* (MW25) showed the highest biosorption capacity with a removal of 66–68% while the remaining *Trichoderma* isolates—namely, *T. asperellum* (S08), *T. asperellum* (LL14), and *T. virens* (SF22)—gave the lowest nickel removal (20–30%).

## 4. Discussion

Our study revealed the isolation of four *Trichoderma* species from different terrestrial and marine substrates, all showing tolerance to nickel as shown by their growth at varying concentrations of this heavy metal in the culture media. Six promising isolates—namely, *T. asperellum* (S03), *T. asperellum* (S08), *T. asperellum* (LL14), *T. virens* (SG18), *T. virens* (SF22), and *T. inhamatum* (MW25)—were further tested and showed the capacity to absorb nickel contaminants under liquid culture. It is also worth mentioning that the terrestrial substrates had the highest number of *Trichoderma* isolates than marine substrates. This is of course expected, as the genus *Trichoderma* is widely isolated from the soil of different grassland and forest ecosystems [32,33], though it has also been isolated from the marine environment [34]. Some of the substrates colonized by many marine-derived fungi, including marine isolates of *Trichoderma*, were seagrasses and seaweeds [35,36,37], with many of these marine-derived fungal strains producing metabolites similar to those observed in terrestrial strains [38]. The successful colonization of many substrata by fungi could be attributed to its ability to degrade different carbon sources [39,40,41].

The genus *Trichoderma* is a large group of fungi that is divided into five sections: *Trichoderma*, *Pachybasium*, *Longibrachiatum*, *Hypocreanum*, and *Saturnisporum* [42], although this number may further change as its phylogeny is resolved and more taxa are discovered [43,44]. Among these sections, our isolates mainly belong to the sections *Trichoderma* and *Pachybasium* (Figure 2). Interestingly, we isolated *T. inhamatum* from marine water (Table 1). This species has been previously reported from soil [45] and known to produce xylanases [46,47] and remove hexavalent chromium [48]. Therefore, it would be an interesting future study to compare the biotechnological applications of marine and terrestrial strains. The most frequently isolated species was *T. asperellum* with 12 isolates, mainly from terrestrial substrates. This species has also been widely reported from the soil in Southeast Asia [49]. Similarly, *T. harzianum* and *T. virens* were also previously recorded from soil samples [45,49] but were isolated in this study from leaf litter and the marine substrates seagrass, seaweed, and sea foam. All our identified *Trichoderma* species follow the valid names [50].

While the *Trichoderma* species listed in this study are known to be of terrestrial origin, we recorded three species—namely, *T. harzianum*, *T. inhamatum*, and *T. virens*—from marine substrates. This is not surprising, as we have previously isolated *Trichoderma* species from marine habitats [36,37]. Marine-derived *Trichoderma* have also been reported by other studies [34] and represent promising sources of bioactive secondary metabolites [51,52]. As observed in this study, the favorable growth of the isolated *Trichoderma* strains in TSM and MEA added with marine salts demonstrated the adaptability of the *Trichoderma* in a marine environment (Figure 3). Marine waters can also influence *Trichoderma* to produce different bioactive metabolites [53,54]. While this study did not look at secondary metabolites, we mentioned these studies to highlight the unique properties that the marine strains of *Trichoderma* have over their terrestrial counterparts. In addition, their occurrence at sea supports the potential use of *Trichoderma* spp. for bioremediation in a marine environment, as it has been observed with other fungi that were isolated from either marine or freshwater habitats and tested for bioremediation in the same aquatic environment [8,9,55].

Looking now at the tolerance and biosorption potential of the *Trichoderma* isolates, *T. asperellum* (S03) isolated from soil exhibited the best growth after two days of incubation even at 1200 ppm of nickel (Table 2). Owing to their ubiquitous distribution, *Trichoderma* species may find application in the bioremediation of pollutants. True enough, previous studies listed several hydrocarbons and heavy metal pollutants that can be cleaned up by different species of *Trichoderma* [11,14]. Specifically, *T. asperellum* and *T. harzianum*, the same species reported herein, were reported to significantly change the chemical composition and structure of used engine oil [15]. *Trichoderma* has also been reported in the uptake of heavy metal [18]. Alginate-immobilized *T. asperellum* has been used in the removal of copper [56]. Therefore, it is not surprising that our terrestrial- and marine-derived Trichoderma could tolerate nickel, grow in its presence, and even absorb it in its hyphae. However, different strains and species showed varied responses (Table 2). Good growth (=highly tolerant strains) to very good growth (=most tolerant strains) were observed as expected with lower Ni concentrations, from 50 to 100 ppm and even up to 500 ppm. From 700 ppm up to 1200 ppm, we still observed a moderate tolerance but with three strains failing to grow at higher concentrations. Filamentous fungi from freshwater ecosystems also showed a tolerance and growth of heavy metals even up to 5000 ppm [57], although, in the study of Nongmaithem et al. [17], their Trichoderma strains could tolerate up to 200-ppm nickel. Interestingly, nickel also affects fungal growth patterns. In this study, we observed changes in the colonial appearance, as well as some modifications in hyphal and spore morphologies. The morphological changes induced by heavy metals are common among fungi. Changes in the mycelial morphology, i.e., curling of hyphae and formation of hyphal coils, were observed in the dark-septate fungus *Gaeumannomyces* *cylindrosporus* in response to Pb [58]. Our study showed a tight aggregation of conidia and the presence of an extensive sheath surrounding the conidia and some disrupted spores.

Fungal biomass has been used in the biosorption of heavy metals. For example, live and dead biomass of *Mucor rouxii* were treated with different heavy metals at different pH [59]. In that study, the live biomass had a higher biosorption capacity than the dead biomass, and this is greatly influenced by the pH. In our study, the *Trichoderma* live biomass removed up to 68% of nickel from the culture medium, albeit the removal capacity varied between strains and species (Table 3). In contrast to our study, in the paper of Hoseinzadeh and colleagues [60], *T. asperellum* showed a better uptake capacity of 78% at 200-mg/L nickel than the 68% at 50 mg/L in our study. They also found the effects of pH and temperature on the uptake of heavy metals by the fungi, with increasing pH values offering a better nickel uptake (highest at pH 8) and a maximum uptake observed at 35 °C. Interestingly, in another study, the nickel biosorption capacity of *T. viride* was better at pH 2 to pH 4.5 [61]. *Trichoderma harzianum* had also the best uptake of nickel at pH 4.5 and temperature of 30 °C [62], though the biosorption of nickel at pH 10 by a dried biomass of *Trichoderma* was observed to be 20% higher than at pH 2 [63]. Therefore, there is a further need to understand what culture conditions are needed to maximize the biosorption capacity of our marine and terrestrial *Trichoderma*. It would be an interesting line of future investigations to look at how the nutrient content of the culture media (e.g., carbon:nitrogen ratio) and other physicochemical parameters such as pH, temperature, aeration, light exposure, and particularly, salinity affects the biosorption or removal capacity of *Trichoderma*, especially the marine isolates. Additional experiments are also needed to fully understand the potential application of *Trichoderma* for the bioremediation of heavy metals, e.g., measuring the metal removal per gram of the fungal biomass, comparing the efficiency of the live versus the dead fungal biomass, and determining the effects of the additional growth supplements on the fungal growth and biosorption capacity.

## 5. Conclusions

Terrestrial and marine substrates were used for the isolation of *Trichoderma*. While strains isolated from marine substrates almost grow comparatively well in the presence and absence of marine salts in their culture media, their growth under saline conditions showed the adaptability of *Trichoderma* in the marine environment. The isolated *Trichoderma* strains showed a tolerance of nickel from 50 ppm up to 1200 ppm, which, however, varies between species and individual isolates. High concentrations of nickel can alter the colony color, while lower concentrations showed little to no changes on the surface morphology of the hyphae. Biosorption of nickel by marine and terrestrial *Trichoderma* strains was also demonstrated, with the percent removal reaching up to 68%. The results of the study proved that *Trichoderma* strains associated with terrestrial and marine substrates are exceptional microorganisms that can tolerate heavy metals and can be explored as bioremediation agents for heavy metal pollution.

## Figures and Tables

**Table 1:** *Trichoderma* isolated from marine and terrestrial substrates collected from Luzon Island, Philippines.

Habitats	Substrates	Taxa	Total Number of Isolates
Terrestrial	Soil	*Trichoderma asperellum*	9
	Leaf Litter	*Trichoderma asperellum* *Trichoderma harzianum* *Trichoderma virens*	3 2 1
Marine	Seagrass	*Trichoderma virens*	1
	Seaweed	*Trichoderma harzianum*	2
	Sea foam	*Trichoderma virens* *Trichoderma harzianum* *Trichoderma harzianum*	1 2 1
	Marine water	*Trichoderma inhamatum*	1

**Table 2:** Tolerance assay of *Trichoderma* strains at different concentrations of nickel.

*Trichoderma* Strains	Nickel Tolerance of Isolated *Trichoderma* at Different Concentrations (ppm)						
	50	100	300	500	700	900	1200
*T. harzianum* LL01	++	++	+	+	−	−	−
*T. asperellum* S02	++	++	++	++	+	+	+
*T. asperellum* S03	+++	+++	++	++	++	+	+
*T. asperellum* S04	++	++	++	++	++	+	+
*T. virens* LL05	+++	+++	++	++	++	+	+
*T. asperellum* LL06	++	++	++	++	+	+	+
*T. harzianum* LL07	++	++	+	+	+	−	−
*T. asperellum* S08	++	++	++	++	+	+	+
*T. asperellum* S09	++	++	++	++	++	+	+
*T. asperellum* S10	++	++	++	++	+	+	+
*T. asperellum* S11	++	++	++	++	+	+	+
*T. asperellum* S12	++	++	++	++	+	+	+
*T. asperellum* S13	++	++	++	++	++	+	+
*T. asperellum* LL14	++	++	++	++	++	+	+
*T. asperellum* LL15	++	++	++	++	+	+	+
*T. harzianum* AL16	++	++	+	+	−	−	−
*T. virens* SW17	++	++	++	++	−	+	−
*T. virens* SG18	++	+++	++	++	++	+	+
*T. harzianum* SF19	++	++	++	+	++	+	+
*T. harzianum* SF21	++	++	+	+	++	+	+
*T. virens* SF22	++	++	++	+	++	+	+
*T. harzianum* SF23	+++	+++	++	++	++	+	+
*T. inhamatum* MW25	+++	++	++	++	++	+	+

Index of Tolerance, Ti: (−) 0 mm, nontolerant; (+) 0.1–0.52 mm, moderately tolerant; (++) 0.53–1.04 mm, highly tolerant; and (+++) 1.05–1.56 mm, most tolerant. The substrate codes were as follows: leaf litter, ground (LL), soil (S), seaweed (SW), seagrass (SG), sea foam (SF), marine water (MW), and leaf litter, aerial or above ground (AL).

**Table 3:** Nickel uptake efficiency of the six *Trichoderma* isolated from marine and terrestrial substrates.

*Trichoderma* Strains ^a^	Absorption of Nickel (NiSO_4_) by *Trichoderma* Biomass	
	Conc. (ppm)	Percent Removal (%)
*T. asperellum* (S03)	16.86	66.28
*T. asperellum* (S08)	39.46	21.08
*T. asperellum* (LL14)	39.79	20.42
*T. virens* (SG18)	15.76	68.48
*T. virens* (SF22)	35.49	29.02
T. inhamatum (MW25)	15.86	68.28

^a^ Significant differences were observed at the 95% confidence intervals (*p* value < 0.05) using a *t*-test.
